# 2-Naphthol Levels and Allergic Disorders in Children

**DOI:** 10.3390/ijerph15071449

**Published:** 2018-07-09

**Authors:** Tien-Jen Lin, Yueliang Leon Guo, Jiin-Chyr Hsu, I-Jen Wang

**Affiliations:** 1 Department of Neurosurgery, Wan Fang Hospital, Taipei Medical University, Taipei 116, Taiwan; trlin1@hotmail.com; 2 Graduate Institute of Sports Science, College of Exercise and Health Sciences, National Taiwan Sport University, Taoyuan 333, Taiwan; 3 Graduate Institute of Injury Prevention and Control, Taipei Medical University, Taipei 110, Taiwan; 4 National Institute of Environmental Health Sciences, National Health Research Institutes, Miaoli 350, Taiwan; leonguo@ntu.edu.tw; 5 Institute of Occupational Medicine and Industrial Hygiene, National Taiwan University College of Public Health, Taipei 100, Taiwan; 6 Department of Internal Medicine, Taipei Hospital, Ministry of Health and Welfare, No. 127, Su-Yuan Road, Hsin-Chuang Dist., Taipei 242, Taiwan; d94841002@ntu.edu.tw; 7 Yuanpei University of Medical Technology, Hsinchu 300, Taiwan; 8 Department of Pediatrics, Taipei Hospital, Ministry of Health and Welfare, No. 127, Su-Yuan Road, Hsin-Chuang Dist., Taipei 242, Taiwan; 9 College of Public Health, China Medical University, Taichung 404, Taiwan; 10 School of Medicine, National Yang-Ming University, Taipei 112, Taiwan

**Keywords:** *naphthol*, children, allergic diseases, oxidative stress

## Abstract

**Background**: The measurement of polycyclic aromatic hydrocarbons (PAH) in ambient air is quite difficult to perform. Using urine biomarkers of PAH such as 2-*naphthol* is one approach to this problem. This study explored the association between urine 2-*naphthol* levels and allergic diseases. The associations between 2-*naphthol* levels and oxidative stress biomarkers for the possible disease pathogenesis were also investigated. **Method**: A total of 453 kindergarten children from the (Childhood Environment and Allergic Diseases Study) CEAS cohort with urine samples were recruited. Urine 2-*naphthol* levels were measured by high-performance liquid chromatography mass spectrometry (HPLC-MS/MS) and markers of oxidative stress (8OHdG) were measured by enzyme-linked immunosorbent assays (ELISA). Information on environmental risk factors and allergic diseases were also collected. The association between 2-*naphthol* levels, 8OHdG levels, IgE, and allergic diseases were evaluated by multivariate linear regression and logistic regression. **Results**: Levels of 2-*naphthol* were positively correlated with 8OHdG levels. A one ln-unit increase in the 2-*naphthol* level was positively associated to 8OHdG levels (per ln-unit: β = 100.61, *p* < 0.001). When dividing 2-*naphthol* levels into quartiles, asthma was significantly associated with 2-*naphthol* levels at a concentration of >1.60 ng/mL (adjusted OR: 3.14, 95% CI 1.34–7.35). **Conclusion**: Urine 2-*naphthol* levels are associated with markers of oxidative stress and the risk of allergic diseases in young children.

## 1. Introduction

Allergic sensitization and asthma have always been associated with exposure to polycyclic aromatic hydrocarbons (PAH) [1,2]. This relationship between environmental exposure to polycyclic aromatic hydrocarbons and the increased risk of incurring allergic disease including asthma in children has been suggested by several epidemiologic studies [3,4]. Data regarding the connection between an individual’s exposure to PAHs and the incidence of pediatric asthma in children are still scarce. Incomplete combustion of hydrocarbons from industrial, domestic, and natural bases is considered as the key source of environmental naphthalene, a polycyclic aromatic hydrocarbon [5,6]. This abundant environmental contaminant is sometimes connected with environmental tobacco smoke (ETS) which is also known as an important indoor pollution source containing various toxic compounds [7]. Individuals without specific occupational exposures are prone to be affected by this environmental naphthalene released from background combustion sources along with cigarette smoke [8]. Oral, dermal, and respiratory routes are considered to be the source of children’s exposure to PAH [8]. Higher levels of PAH metabolites were found amongst children, indicating a potential higher risk for this population [8,9]. Nevertheless, the relationships between this scale of PAH metabolite levels and allergy-related disease outcomes have never been evaluated.

Naphthalene is broken down by cytochrome P450 enzymes to form naphthalene oxide which is reorganized to 1- and 2-*naphthol* after being absorbed by inhalation or ingestion [10]. Some recent investigations indicated that 2-*naphthol* should be considered a more specific biomarker of exposure for aerosol PAH [11,12]. Furthermore, 2-*naphthol* can induce oxidative/nitrative stress in vitro and in vivo by generating reactive oxygen and nitrogen species (ROS/RNS) as shown by several recent studies [13]. Promotion of tissue inflammation through upregulation of genes that code for pro-inflammatory cytokines can be evoked by oxidative stress. In turn, inflammatory cells release free radicals when activated. The role of oxidative stress in the pathogenesis of allergic disorders is generally conceivable given that its prominent inflammatory component. Consequently, the most important biomarker of oxidative/nitrative stress for free radical-mediated oxidative/nitrative products of DNA and lipids is currently considered to be 8-hydroxy-2′-deoxyguanosine (8-OHdG). The degree of oxidative damage has been quantified most often by measuring urinary 8-OHdG using enzyme-linked immunosorbent assays (ELISA), particularly because this technique is non-invasive [14].

Very little information is available on biomarkers that reflect oxidative stress induced by PAH exposure and their connection to allergic disease in pediatric populations. The level of 2-*naphthol* in urine is used to estimate levels of DNA damage in sperm of occupationally-exposed agricultural workers [15]. Noticeably lower serum IgA levels (geometric mean exposure vs. no exposure: 1289 mg/dl vs. 1551 mg/dl, *p* < 0.001) and IgG levels (geometric mean exposure vs. no exposure: 230 mg/dl vs. 276 mg/dl, *p* < 0.001) and slightly elevated serum IgE levels (geometric mean exposure vs. no exposure: 140 IU/dl vs. 80 IU/dl) were seen among coke oven workers exposed to PAHs when compared to workers with no PAH exposure [16]. Based on current epidemiological data, information about immunological changes after chronic occupational exposure to PAHs is scarce [17]. Until now, biomarkers linked to PAH exposure and its connection to oxidative stress related allergic disease have still been under investigation [17].

Few studies have even examined the risk factors for environmental PAH exposure. Higher incidences of hospital admissions, chronic asthma, and premature death, along with respiratory problems in children are considered to be associated with daily exposure to PAH contaminated particulate substance [18]. Due to the immaturity of their organs and increased dosage per unit body surface area, young children are more susceptible to toxic chemicals. Under such circumstances, this study was intended to investigate (i) the association between urinary 2-*naphthol* levels and allergic diseases in children; (ii) the association between urinary 2-*naphthol* levels and biomarkers of DNA damage (8-OHdG levels) for the possible disease pathogenesis in connection with oxidative stress; and (iii) the association between 8-OHdG (i.e., oxidative stress) and allergic diseases.

## 2. Methods

### 2.1. Study Population

A total of 453 kindergarten children with available urine and blood specimens were selected from the 3246 Childhood Environment and Allergic Diseases Study (CEAS) cohort in 2010 in Taiwan [19]. There were no significant differences between the original cohort and the final participants. The concentrations of urinary 2-*naphthol* were measured at 3 years of age as an indicator of PAH exposure and serum total IgE levels were determined at the same age as an indicator of sensitization. We also collected basic data on the children’s allergic diseases to determine the association between 2-*naphthol* levels and allergic disorders. We also acquired written informed consent from all the parents in compliance with the principles of the Helsinki Declaration. The Taipei hospital’s Institutional Review Board ratified the study protocol (TH-IRB-0015-0029).

### 2.2. Allergic Diseases

A formalized history and clinical examination of participants with parental-reported doctor-diagnosed atopic dermatitis (AD), allergic rhinitis (AR), and asthma from the ISAAC questionnaire were achieved at outpatient clinics. The cases with AD were further proved according to the diagnostic criteria developed by Hanifin and Rajka [20], while AR was further confirmed by the Allergic Rhinitis and its Impact on Asthma (ARIA) guidelines [21]. Asthma was determined by pediatric allergists based on one of the following three criteria: (i) recurrence of at least two of the three symptoms: cough, wheeze, and shortness of breath within the previous 1 year without having a cold, (ii) doctor’s diagnosis of asthma with ongoing treatment, (iii) response to treatment after β2-agonists or inhaled corticosteroid use [22]. Patients visiting the clinic without fulfilling the above criteria were defined as non-allergic subjects.

### 2.3. Laboratory Methods

#### 2.3.1. Urine Analyses

The first mid-stream urine in the morning was collected from children at 3 years of age and stored at −20 °C until analysis. Frozen urine samples were thawed at 4 °C and then vortexed for 30 s to achieve homogeneity. The sample preparation methods used on this study were based on Kim et al. (1999) [12]. A 400 µL urine sample was placed into a polypropylene centrifuge tube and then vortexed with 40 µL of 2 M sodium acetate (pH 5.0) (Merck, Darmstadt, Germany) for 30 s. Then 5 µL of β-glucuronidase with sulfatase (*Helix pomatia*, H2, G0876) (Sigma-Aldrich Co., St. Louis, MO, USA) and 20 µL of 1 µg/mL internal standard solution of 2-*naphthol*-d7 (Sigma-Aldrich Co., St. Louis, MO, USA) was added to each sample before the second vortex in dark room. The samples were incubated at 37 °C overnight for 16 h. After the incubation, the deconjugated urine samples were diluted with 800 µL of acetonitrile (ACN) (HPLC grade, J.T.Baker) and then vortexed with 80 µL of 20 mM formic acid (J.T.Baker) (pH 2.7). The samples were centrifuged at 6000 rpm at 4 °C for 10 min and the supernatant was collected. Finally, the samples were filtered through a 0.22 µm polyvinylidene difluoride (PVDF) syringe filter into a screw cap vial.

Levels of 2-*naphthol* were measured using liquid chromatography-tandem mass spectrometry (LC-MS/MS) system with isotope-dilution. The LC-MSMS system used in this study was comprised of an Agilent 1100 series system (Agilent Tech., Santa Clara, CA, USA) coupled to a Finnigan TSQ Quantum Discovery Max spectrometer system (Thermo Electron Corporation, Breda, The Netherlands) with an electron spray ionization (ESI) source in negative ion mode. The sample (20 µL) was injected onto a 2.0 mm × 100 mm Capcell Pak^®^ 3 µm C18 column (SHISEIDO Co., Tokyo, Japan) with mobile phases consisting of 10 mM ammonium acetate (Sigma-Aldrich Co., St. Louis, MO, USA) in LC-MS grade water (Scharlab, Barcelona, Spain) and pure ACN was delivered at a constant flow rate of 0.25 mL/min. The following linear gradient for elution was applied: initially eluted in 40% ACN, linearly increased to 90% ACN after 2 min, retained for 8 min in 90% ACN, returned to 40% ACN after 12 min, and held in 40% can for 6 min till the end of the run. Optimized MS parameters were as follows: spray ion voltage of 3700 V, capillary temperature of 250 °C, sheath gas pressure of 35 arbitrary units, auxiliary gas pressure of 10 arbitrary units, collision gas pressure of 1.0 mTorr, and dwell time of 100 ms. The detection was carried out in selective reaction monitoring (SRM) mode. The collision energy (V) and SRM transitions monitored were as follows: 25 V, *m*/*z* 143→115 for 2-*naphthol*; 34 V, *m*/*z* 150→122 for 2-*naphthol*-d7. The limit of detection (LOD) was 0.145 ng/mL. A value of half the limit of detection was assigned for concentrations below the detection limit. Urine 8-OHdG was analyzed using an enzymatic assay (Cayman Chemical Company, Ann Arbor, MI, USA), with a LOD of 0.32 ng/mL. Urine creatinine levels were also measured using an enzymatic assay (Cayman Chemical, Ann Arbor, MI, USA) (Cayman Chemical Company 2012). All experiments were done in duplicate. In the statistical analysis, all 2-*naphthol* and 8-OHdG levels were adjusted for urine creatinine levels.

#### 2.3.2. Total IgE Analysis

Serum total IgE levels were collected at 3 years of age and measured by the Pharmacia UniCap IgE assay test system according to the manufacturer’s prescribed protocol (Pharmacia Diagnostics, Uppsala, Sweden). Concentrations below 0.35 kU/L were defined as undetectable IgE. Total IgE levels were considered increased at values of greater than 100 kU/L [23].

### 2.4. Statistical Analysis

The study of urine 2-*naphthol* levels across different characteristics was performed using two sample t-test. The associations between urine 2-*naphthol* and IgE levels and between urine 2-*naphthol* and 8-OHdG levels were analyzed by linear regression. The relationships between urine 2-*naphthol* and allergic diseases were analyzed by univariate and multivariate logistic regression. Data with skewed distributions were log (Ln)-transformed before further analyses. All log-transformed data in the study had a normal distribution and no significant outliers were found. Potential confounders from a review of the literature, including gender, gestational age, parity, maternal age, education, occupation, diets and supplements during pregnancy, family income, parental atopy, duration of breast feeding, fungi on house walls, tobacco smoke exposure, and incensing and carpets at home were all taken into consideration. Only those potential confounders with a 10% change in point estimate of the crude model were included in the final model. All hypothesis testing was two-sided and statistical significance was set at *p* < 0.05. All analyses were performed using the SAS software version 9.1 (SAS Institute, Inc., Cary, NC, USA).

## 3. Results

Initially, 453 children were enrolled. The data of the study population basic demographics is provided in Table 1. There were 11.7% (53/453) of the children with AD, 31.8% (144/453) with AR and 26% (118/453) with asthma. The median (range) of IgE levels and 8OHdG levels were 113.0 (2364.10) (kU/L) and 19.37 (2643.81) (ng/mL). The geometric means (GM) (geometric standard deviation, GSD) of 2-*naphthol* concentrations were 11.84 (3.35) µg/g creatinine (Table S1). A summary of means of urine 2-*naphthol* concentrations in relation to the characteristics of mothers and children was outlined in Table 2. Socioeconomic risk factors such as lower maternal education may be associated with higher 2-*naphthol* levels (Mean ± SD 4.06 (15.41) vs. 1.71 (3.49), *p* = 0.03). Boys, prematurity, low birth weight, young maternal age, and low family income may also be associated with higher 2-*naphthol* levels, though analyses failed to reach statistical significance. Other demographic characteristics were not statistically significant.

### 3.1. Association between 2-Naphthol Levels and Total IgE Levels

There was no significant correlation between 2-*naphthol* concentrations and IgE levels, as shown in Table 3.

### 3.2. Association between 2-Naphthol Levels and 8OHdG Levels

Table 4 showed the regression coefficients β (95% CI) for urine 8OHdG according to the ln-*naphthol* concentrations. One ln-unit increase of urinary 2-*naphthol* level was positively associated with 8OHdG levels (β = 100.61, *p* < 0.001) after adjusting for potential confounders. Analyses stratified by gender revealed no significant gender difference. In our study, the association between 2-*naphthol* exposure and both 8OHdG and IgE was higher in females than in males.

### 3.3. Association between 2-Naphthol Levels and Allergic Diseases

Levels of 2-*naphthol* were significantly increased in asthmatic individuals compared to non-asthma control subjects (GM (GSD): 1.32 (1.08) vs. 0.82 (1.12) (ng/mL)). Levels of 2-*naphthol* were significantly increased in AR individuals compared to non-AR control subjects (GM (GSD): (1.19 (1.10)) vs. (0.81 (1.08)) (ng/mL)). Levels of 2-*naphthol* were significantly increased in AD individuals compared to non-AD control subjects (GM (GSD): 1.49 (1.21) vs. 0.86 (1.06) (ng/mL)). When dividing 2-*naphthol* levels into quartiles, there was a significant relationship between 2-*naphthol* levels and asthma (adjusted OR 3.14, 95% CI 1.34–7.35) as shown in Table 5. AR and AD were positively associated with 2-*naphthol* levels, though these failed to reach statistical significance. Analyses stratified by gender revealed that there was no gender difference, as shown in Table 5.

### 3.4. Associations between 8OHdG and Allergic Diseases

When dividing 8-OHdG levels by median levels, allergic diseases including asthma, allergic rhinitis and AD were positively associated with urinary 8-OHdG levels. These results reached statistical significance, as shown in Table 6.

## 4. Discussion

This study highlights potential associations between 2-*naphthol* levels, oxidative stress, and outcomes of childhood allergic disorders. We also found that urinary levels of 8-OHdGs were associated with 2-*naphthol* levels. These findings not only help in understanding the role of PAH exposures in developing allergies, but also generate potential monitoring measures for susceptible populations.

### 4.1. Exposure to PAH and Socio-Economic Risks in the Development of Allergic Disorders

The main urban source of PAHs is usually traffic, although in highly industrial areas, traffic pollution may be ancillary [24]. Particulate matter 2.5 (PM_2.5_) and PM_2.5_-bound PAHs occurring in both outdoor and indoor air are the main source of children’s exposure to PAHs at the kindergartens in the Silesian region of Poland [25]. In a recent survey in Taiwan, concentration of exposure to PAHs in residential indoor environments was found to be a geometric mean of total gaseous and particulate PAHs of 267 ng/m^3^ [26]. As in Lithuanian primary schools, equivalent concentrations were seen (Σ15 PAHs range 20.3–131.1 ng/m^3^) [27]. By comparison, concentrations of the sum of 12 selected PAHs in kindergartens in Montreal were lower both in polluted (3.0 ng/m^3^) and control areas (2.1 ng/m^3^) [9]. Concentrations of the sum of 16 PAHs were measured to be 1.4 and 4.2 ng/m^3^ in two Portugal kindergartens interior [28]. Our results showed that maternal education had a significant effect on 2-*naphthol* levels in children. However, there are few reports of the relationship of maternal education and 2-*naphthol* levels in the literature review. After adjusting for maternal education, the relationship between 2-*naphthol* levels and allergic diseases remained statistically significant.

In our studied subjects, their exposure to PAHs originated from multiple sources in the urban area of Northern Taiwan. The most featured indoor pollution source is known to be environmental tobacco smoke (ETS). In certain public places such as schools, libraries, hospitals, and also on public transportation in Taiwan, smoking is prohibited by law. ETS cannot be regarded as the primary source of PAHs in our study suggested by the following reasons: first, results of our investigation showed that urinary levels of 2-*naphthol* in both non-ETS and ETS are at comparably similar concentrations. Second, roadside and traffic PAH concentrations are higher in urban locations, which sequentially are higher than rural ones (European Environment Agency (EFA), 2012). Third, most of our studied subjects dwell within the metropolitan area in Northern Taiwan. In a typical Taiwanese residential area, total gaseous and particulate PAHs detected in both indoor and outdoor environments is comparably high due to heavy traffic exhaust emissions [26]. Fourth, most PAH exposures for non-occupationally exposed individuals occur primarily indoors at home [26]. Most of our studied subjects are pre-school children. At present, there is no direct evidence linking allergic diseases and 2-*naphthol* levels with exposure to roadside traffic. However, in our previous study, we found that PM_2.5_ concentrations (also highly connected with roadside traffic) have certain correlation with allergic diseases. We also found that PM_2.5_, PM_10_, and CO were significantly associated with asthma [29]. Bae et al*.* also indicated that exposure to PM air pollution and PAHs were associated with oxidative stress in school children [30]. Therefore, we highly suspect the primary source of PAH exposure of our studied subjects to be gaseous and particulate PAHs emitted from roadside traffics.

### 4.2. Association between Urinary 2-Naphthol Levels and Allergic Diseases in Children

The level of 2-*naphthol*, compared to that of 1-*naphthol*, in urine was reported to reflect more specifically the exposure to PAHs in ambient air [11,12]. Contact to PAHs is known to provoke induction of reactive oxygen species (ROS) and consequent lipid peroxidation or DNA damage [31,32]. In recent years, 8-OHdG has emerged as a marker of oxidative stress causing lipid peroxidation or DNA damage [33]. Excessive ROS/RNS are synthesized in the process of oxidative metabolism; they are also considered to be inflammatory stimuli reported to initiate the inflammatory process resulting in production and secretion of pro-inflammatory cytokines [34,35]. Sequentially, inflammatory cells give out free radicals when activated. It is believed that oxidative stress may play a role in the pathogenesis of allergic disorders due to their prominent inflammatory component. Different levels of 2-*naphthol* are identified to be higher in asthmatics compared to non-asthma control subjects. The geometric means (s.d.) of *naphthol* concentrations were 11.84 (3.35) µg/g creatinine in our study which were significantly higher compared to studied subjects in other series [18,36]. In this study, there was no positive association between 2-*naphthol* and other allergic diseases besides asthma. Therefore, it is suggested that exposure to PAH in the ambient air was the primary source of allergens in these studied subjects.

### 4.3. Association between Urinary 2-Naphthol Levels and 8-OHdG for the Possible Disease Pathogenesis of Asthma

The present study showed positive associations between 2-*naphthol* concentration and 8-OHdG levels. Our results also demonstrated that urinary 2-*naphthol* level was positively correlated with asthma in pre-school children. However, there was no positive association between 2-*naphthol* and IgE levels. The result was consistent with previous in vitro and animal studies showing that 2-*naphthol* can act as an allergic sensitizer by generating reactive oxygen species (ROS) induction responses [37,38]. Urinary 8-OHdG levels were also significantly related with sensitization to various specific allergens in a dose-response manner. Based on our current understanding, it is theorised that asthma is associated with high levels of oxidative stress which is a consequence of both elevated oxidant forces and reduced antioxidant capacity [39]. Increased incidence of bronchial asthma has also been reported in areas with high levels of air pollution [3]. Likewise, PAH exposure can also cause rigorous asthma and frequent asthma attacks [3]. However, it is still uncertain whether amplified oxidative stress in the asthmatic airway is merely an outcome of chronic airway inflammation or a vital causal factor in the process of allergic inflammation. Based on our findings in this study, we suggest that increased oxidative stress may play a critical role in mounting an inflammatory response in pre-school children with asthma caused by PAH exposure with their consequent higher urinary levels of 8-OHdG.

### 4.4. Allergic Disorders due to PAH Exposure is Associated with Oxidative Stress and Its Related Biomarkers

In environmental epidemiology, exposure biomarkers are valuable for monitoring internal dose that justifys all means of exposure. Non-invasive sampling by simple urine collection to quantify substances or metabolites makes biological monitoring valid for large numbers of samples. However, to interpret the data, a suitable reference range would be helpful. PAHs levels were significantly higher in asthmatic individuals compared to non-asthma control subjects as shown in past literature [40]. Since there are limited data available in non-occupationally exposed individuals for the PAH concentrations in the environment, conjugates of 1-*naphthol* and 2-*naphthol* in urine are considered to be associated with this toxic exposure [15]. Environmental and dietary exposures to naphthalene are generally reflected in background population levels of 1-*naphthol* [15]. Based on the current consensus view, the more specific biomarker of exposure to aerosol PAH is thought to be the presence of 2-*naphthol* in a urine sample which is also been identified as exposure to naphthalene [15]. Contact with PAHs was also related to oxidative stress in school children [30]. Effectiveness of dietary supplements with regard to reduction in oxidative damage and monitoring of oxidative stress in the studied subjects has also been evaluated with urinary 8-OHdG [41]. Our results demonstrated that 2-*naphthol*, considered a marker of exposure to naphthalene in children, is associated with oxidative stress and related to allergic disorders such as asthma. Therefore, we suggest that urinary 2-*naphthol* levels can be used as a biomarker to monitor PAH exposure in the air causing allergic diseases such as asthma.

## 5. Limitations and Strengths

Several limitations should be acknowledged. First, the study is a cross-sectional survey of the relationship between urinary levels of 2-*naphthol* in reflection to PAHs exposure and asthma occurrence. A future longitudinal follow up will be needed to validate the accuracy of this urinary biomarker of PAHs exposure. Second, we used spot urinary 2-*naphthol* levels as an alternative for exposure. Significant day-to-day variations in exposure to PAHs may be problematic as such. Sobus et al. collected pre-, post-shift, bedtime and morning samples from 20 asphalt pavers; the calculated half-lives were 26 h (95% CI: 14–116 h) for *naphthols* (summation of 1 and 2-*naphthol*) [42]. St. Helen et al. followed 8 smokers and investigated excretion kinetics of urinary PAHs after cigarette smoking; the average half-lives were 9.4 h (4.9–12.2 h) for 2-*naphthol* [43]. Measurements in spot urine samples may adequately reflect the exposure of the average population when sample sizes are large, and half-life of the metabolites are prolonged, even though they are often limited by poor intra-class associations coefficients [43,44]. We also consider the population-based design and exposure assessment of objective biomarkers to be strengths of our study.

## 6. Conclusions

Our data suggested that urine 2-*naphthol* levels were associated with markers of oxidative stress and the risk of allergic diseases in children. Studies relating exposure to PAH as potentially associated with oxidative stress and asthma-related symptoms are scarcely seen in current literature. Our results indicate that more work is needed to discover a clinically useful, non-invasive monitoring for progress and treatment of allergic diseases.

## Figures and Tables

**Table 1:** Demographics of the study population.

			Asthma			AR			AD	
Characteristic	Total	Asthma	Non-Asthma	*p*-Value	AR	Non-AR	*p*-Value	AD	Non-AD	*p*-Value
Total number	453	118	335		144	309		53	400	
Mother										
Maternal age (years old)										
Mean ± SD	29.39 ± 4.4	29.62 ± 4.1	29.31 ± 4.5	0.64	29.37 ± 4.52	39.40 ± 4.29	0.41	29.54 (3.49)	29.47 (4.47)	0.065
Maternal education (%)										
≥College	118 (26.1)	32 (25.4)	86 (26.3)	0.89	40 (31.8)	78 (23.9)	0.67	15 (28.3)	103 (25.8)	0.86
Maternal history of atopy (%)										
Yes	126 (27.8)	46 (36.5)	80 (24.5)	0.002	56 (38.9)	70 (22.7)	0.001 *	25 (47.2)	101 (25.3)	0.002 *
Children										
Male (%)	187 (41.3)	47 (37.3)	140 (42.8)	0.50	94 (82.5)	156 (50.5)	0.012 *	35 (66.0)	214 (53.5)	0.16
Gestational age (%)										
<37weeks	353 (77.9)	91 (72.2)	262 (80.1)	0.99	111 (77.1)	242 (78.3)	0.73	42 (79.2)	311 (77.8)	0.37
Parity (%)										
<2	315 (69.5)	84 (66.7)	231 (70.6)	0.64	105 (72.9)	210 (68.0)	0.30	41 (77.4)	274 (68.5)	0.10
Environmental factors										
Breast feeding (%)										
Yes	292 (64.4)	75 (59.5)	217 (66.4)	0.42	91 (63.2)	201 (65.1)	0.33	37 (69.8)	255 (63.8)	0.69
Incensing at home (%)										
Yes	220 (48.6)	65 (51.6)	155 (47.4)	0.16	76 (52.8)	144 (46.6)	0.33	27 (50.9)	193 (48.3)	0.84
ETS exposure (%)										
Yes	184 (40.6)	58 (46.0)	126 (38.5)	0.028 *	70(48.6)	114(36.9)	0.028 *	26 (49.1)	158 (39.5)	0.19
Family income per year (NT dollars) (%)										
<600,000	110 (31.3)	25 (29.4)	85 (32.0)	0.80	39 (36.8)	71 (29.0)	0.064	13 (31.7)	97 (31.3)	0.99
600,000–1,500,000	213 (60.7)	52 (61.2)	161 (60.5)		55 (51.9)	158(64.5)		25 (61.0)	188 (60.7)	
>1,500,000	28 (8.0)	8 (9.4)	20 (7.5)		12 (11.3)	16 (6.5)		3 (7.3)	25 (8.1)	

Abbreviations: ETS, environmental tobacco smoke; some numbers do not add up to total *n* because of missing values. * *p* < 0.05.

**Table 2:** Geometric mean (SD) of urine naphthol levels (ng/mL) by characteristics.

Characteristics	Mean ± SD	*p*-Value
*Mother*		
Maternal age		
<34 years	3.74 (14.50)	0.263
≥34 years	1.55 (2.81)	
Maternal education		
<College	4.06 (15.41)	0.030 *
≥College	1.71 (3.49)	
Maternal history of atopy		
No	3.30 (15.28)	0.881
Yes	3.06 (7.77)	
*Children*		
Gender Male	4.89 (20.77)	0.125
Female	2.51 (6.97)	
Birth weight		
<2500 g	3.35 (13.40)	0.533
≥2500 g	1.19 (1.23)	
Gestational age		
<37 weeks	3.28 (13.31)	0.609
≥37 weeks	2.00 (4.86)	
Parity		
<2	3.51 (14.07)	0.571
≥2	2.45 (5.49)	
*Environmental factors*		
Breast feeding		
No	2.65 (5.89)	0.690
Yes	3.33 (14.08)	
Incensing at home		
No	4.46 (18.29)	0.189
Yes	2.38 (5.89)	
ETS exposure		
No	3.04 (11.90)	0.771
Yes	3.44 (13.89)	
Family income per year		
<600,000 NT dollars	4.18 (16.96)	0.599
≥600,000–1,500,000 NT dollars	3.29 (12.21)	
>1,500,000 NT dollars	1.21 (1.18)	

ETS: environmental tobacco smoke; NT: New Taiwan dollars; * *p* < 0.05.

**Table 3:** Regression coefficients β (95% CI) for total IgE (KU/l) according to urine Ln-naphthol levels, stratified by gender.

Total (*n* = 453)	Ln-Naphthol	95% CI	*p*
blood IgE			
Adjusted β ^a^	0.78	(−4.16–2.60)	0.649
Boys (*n* = 262)	Ln-naphthol		*P*
blood IgE			
Adjusted β ^a^	0.16	(−3.69–3.37)	0.926
Girls (*n* = 191)	Ln-naphthol		P
blood IgE			
Adjusted β ^a^	7.36	(−21.19–6.47)	0.286

^a^ Adjusted for urine creatinine, maternal age, maternal education, maternal history of atopy, breast feeding, and ETS exposure.

**Table 4:** Regression coefficients β (95% CI) for urine 8-OHdG according to Ln-naphthol concentrations stratified by gender.

Total (*n* = 453)	Ln-Naphthol	*p*
8-OHdG (ng/mL)		
Adjusted β (95% CI)	100.61 (49.41–151.81)	<0.001 *
Boys (*n* = 261)	Ln-naphthol	
8-OHdG (ng/mL)		
Adjusted β (95% CI) ^a^	104.95 (17.05–192.85)	0.020 *
Girls (*n* = 191)	Ln-naphthol	
8-OhdG (ng/mL)		
Adjusted β (95% CI) ^a^	144.29 (52.32–236.26)	0.002 *

* *p* < 0.05; ^a^ Adjusted for urine creatinine, maternal age, maternal education, maternal history of atopy, breast feeding, and ETS exposure.

**Table 5:** Association between urine naphthol levels (ng/mL) and allergic diseases across four categories.

Total (*n* = 453)		Naphthol			
		<0.47 ng/mL	0.47–0.74 ng/mL	0.74–1.60 ng/mL	>1.60 ng/mL
Asthma Adjusted OR (95% CI) ^a^		1.00	1.32 (0.54–3.24)	1.07 (0.42–2.73)	3.14 (1.34–7.35) *
	Boys	1.00	2.43 (0.67–8.79)	0.82 (0.19–3.66)	4.09 (1.17–14.31) *
	Girls	1.00	0.64 (0.17–2.50)	1.49 (0.44–5.02)	3.34 (1.04–10.71) *
Allergic rhinitis Adjusted OR (95% CI) ^a^		1.00	0.67 (0.30–1.49)	0.93 (0.43–2.01)	1.60 (0.75–3.38)
	Boys	1.00	0.84 (0.30–2.38)	0.77 (0.27–2.21)	1.65 (0.61–4.46)
	Girls	1.00	0.43 (0.11–1.62)	1.08 (0.34–3.43)	1.32 (0.41–4.30)
Atopic dermatitis Adjusted OR (95% CI) ^a^		1.00	0.57 (0.15–2.12)	1.09 (0.34–3.45)	1.88 (0.64–5.51)
	Boys	1.00	0.53 (0.08–3.39)	0.77 (0.14–4.12)	1.69 (0.38–7.52)
	Girls	1.00	0.63 (0.10–4.16)	1.34 (0.26–6.88)	1.98 (0.40–9.73)

* *p* < 0.05; ^a^ Adjusted for urine creatinine, maternal age, maternal education, maternal history of atopy, breast feeding, and ETS exposure.

**Table 6:** Relationships between 8-OHdG and allergic diseases.

Total (*n* = 453)		8-OhdG	
		<17.9056	>17.9056
Asthma Adjusted OR (95% CI) ^a^	total	1.00	10.75 (4.85–23.81) *
Allergic rhinitis Adjusted OR (95% CI) ^a^	total	1.00	11.00 (5.47–22.12) *
Atopic dermatitis Adjusted OR (95% CI) ^a^	total	1.00	16.89 (3.94–72.38) *

* *p* < 0.05; ^a^ Adjusted for urine creatinine, maternal age, maternal education, maternal history of atopy, breast feeding, and ETS exposure.

## Supplementary Materials

The following are available online at http://www.mdpi.com/1660-4601/15/7/1449/s1, Table S1: The distribution of naphthol, IgE and 8-OHdG levels (ng/mL).
