# Supplementary material for: 2-Naphthol Levels and Allergic Disorders in Children
Source: Int J Environ Res Public Health. 2018 Jul 9;15(7):1449. doi: 10.3390/ijerph15071449 (PMC6069002; doi:10.3390/ijerph15071449)
Supplement: Supplementary file 1 [file ijerph-15-01449-s001.pdf]

Supplementary Materials:

Table S1. The distribution of naphthol, IgE and 8-OHdG levels (ng/mL).

	> LOD (%)	Minimum	Maximum	GM (GSD)
Naphthol	96.0	0.073	201	11.84 (3.35)
IgE	97.47	1.90	2366.00	100.64(1.08)
8OH-dG	99.77	0.00	2644.13	75.44(1.11)

LOD: Limit of detection = 0.145 ng/mL; ng/mL; GM: Geometric mean; GSD: geometric standard deviation.
